# Influence of exercise type and duration on cardiorespiratory fitness and muscular strength in post-menopausal women: a systematic review and meta-analysis

**DOI:** 10.3389/fcvm.2023.1190187

**Published:** 2023-05-09

**Authors:** Mousa Khalafi, Mohammad Hossein Sakhaei, Aref Habibi Maleki, Sara K. Rosenkranz, Mohammad Javad Pourvaghar, Yiqun Fang, Mallikarjuna Korivi

**Affiliations:** ^1^ Department of Physical Education and Sport Sciences, Faculty of Humanities, University of Kashan, Kashan, Iran; ^2^ Department of Exercise Physiology, Faculty of Sport Sciences, University of Guilan, Guilan, Iran; ^3^ Department of Exercise Physiology and Corrective Exercises, Faculty of Sport Sciences, Urmia University, Urmia, Iran; ^4^ Department of Kinesiology and Nutrition Sciences, University of Nevada Las Vegas, Las Vegas, NV, United States; ^5^ Department of Emergency, Jinhua Guangfu Oncology Hospital, Jinhua, China; ^6^ Institute of Human Movement and Sports Engineering, College of Physical Education and Health Sciences, Zhejiang Normal University, Jinhua, China

**Keywords:** aerobic training, resistance training, cardiorespiratory fitness, muscular strength, postmenopausal women

## Abstract

**Background and aim:**

Both cardiorespiratory fitness (CRF) and muscular strength are reported to decrease with age and menopause, which considered to be risk for cardiovascular diseases (CVDs). Previous relevant meta-analyses are inconclusive on the beneficial effects of exercise, particularly in post-menopausal women. In this systematic review and meta-analysis, we investigated the effects of exercise modalities on CRF and muscular strength in post-menopausal women, and identified the effective exercise type and duration.

**Methods:**

A comprehensive search was conducted on PubMed, Web of Science, CINAHL, and Medline to identify the randomized controlled trials, which evaluated exercise effect on CRF, lower- and upper-body muscular strength, and/or handgrip strength in post-menopausal women and compared the results with control. Standardized mean differences (SMD), weighted mean differences (WMD), and 95% confidence intervals (95% CIs) were calculated using random effects models.

**Results:**

A total of 129 studies comprising 7,141 post-menopausal women with mean age and BMI ranging from ∼53 to 90 years and 22 to 35 kg/m^2^, respectively, were included in the meta-analysis. Overall, exercise training effectively increased CRF (SMD: 1.15; 95% CI: 0.87, 1.42; *p* = 0.001), lower-body muscular strength (SMD: 1.06; 95% CI: 0.90, 1.22; *p* = 0.001), upper-body muscular strength (SMD: 1.11; 95% CI: 0.91, 1.31; *p* = 0.001), and handgrip strength (WMD: 1.78 kg; 95% CI: 1.24, 2.32; *p* = 0.001) in post-menopausal women. These increments were found to be irrespective of ages and intervention durations. Regarding exercise type, aerobic, resistance, and combined training significantly increased CRF and lower-body muscular strength, while resistance and combined training effectively increased handgrip strength. However, only resistance training increased the upper-body muscular strength in women.

**Conclusion:**

Our findings suggest that exercise training is effective in increasing CRF and muscular strength in post-menopausal women, which might be cardioprotective. Both aerobic and resistance training alone or in combination increased CRF and lower-body muscular strength, but only resistance training increased upper-body strength in women.

**Systematic Review Registration:**

https://www.crd.york.ac.uk/prospero/display_record.php?RecordID=283425, identifier: CRD42021283425.

## Introduction

The post-menopausal stage of life is associated with an increased risk for cardiovascular diseases (CVDs), which might be due to the decreased cardioprotective-estrogens levels that co-occur with decreased physical activity. Cardiorespiratory fitness (CRF), as defined by maximal oxygen uptake (VO_2max_), is an important independent predictor of CVDs, and is related to other risk factors including dyslipidemia, insulin resistance, chronic low-grade inflammation, and hypertension (1–4). It is well established that a progressive decrease in muscular mass and strength, known as sarcopenia, is associated with increased risk for CVDs and all-cause mortality. In addition, these decreases progress with age and rapid progression coincides with the onset of the post-menopausal period (5–8).

Sedentary behavior and insufficient physical activity are the important modifiable risk factors for developing CVDs and CVD-related mortality (9). Lifestyle interventions often emphasized that exercise training can improve cardiometabolic risk factors, and have been proposed as first-line therapeutic strategies. Although exercise and dietary interventions are effective for improving cardiovascular risk factors (dyslipidemia, insulin resistance, chronic low-grade inflammation, and hypertension), the independent effect of exercise on CRF and muscular strength is also superior owing to its beneficial effects in prevention and treatment of CVDs. Exercise training is an effective strategy for increasing CRF and muscular strength across a wide range of participant characteristics. However, previous systematic reviews and meta-analyses suggest that the type of exercise plays an important role in these adaptations. For instance, aerobic training has been shown to improve CRF in older adults (10, 11), while resistance training and combined training have been shown to improve muscular strength in elderly (12–14). On the other hand, combined training is thought to promote both CRF and muscular strength in middle-aged to older adults (15).

However, no comprehensive meta-analyses have been conducted yet on post-menopausal women to address the influence of exercise modalities on CRF and muscle strength. Given the evidence that increased cardiovascular risk during post-menopausal period, and established beneficial effects of exercise in non-menopausal population, we hypothesized that exercise intervention might be more useful among post-menopausal women than other population. Therefore, the present meta-analysis aimed to elucidate the effects of exercise modalities on CRF and muscular strength in post-menopausal women. We then investigated the influential role of “exercise type and exercise duration” on the beneficial effects to suggest the practical and clinical recommendations of exercise for post-menopausal women.

### Methods

The present systematic review and meta-analysis was performed in accordance with the latest guidelines of Preferred Reporting Items for Systematic Reviews and Meta-Analyses (PRISMA), and the Cochrane Handbook of Systematic Reviews of Interventions (16, 17). This systematic review and meta-analysis was registered in the international Prospective Register of Systematic Reviews (PROSPERO-ID: CRD42021283425).

### Search strategy

Four main electronic databases, including PubMed, Web of Science, CINAHL, and Medline were systematically searched from inception to October 2022 using the following key words or search terms: “menopausal” or “post menopause” or “post-menopause” or “menopause” or “elderly women” or “older women” AND “exercise” or “exercise training” or “physical activity”. During search, the Boolean operators “AND” and “OR” were used to combine the key words. The following limitations were applied when available in the databases: English language, human participants, article document type, and randomized controlled trials. The reference lists of previous relevant systematic reviews (18) and all studies included in the current systematic review were manually searched to ensure no relevant articles were missed.

### Study selection and inclusion and exclusion criteria

Article search and selection was conducted by two independent reviewer authors (A H M and M H S), and any disagreements were resolved by discussion with another reviewer (M kh). All studies from the search of databases were exported into Endnote 20. Duplicate publications were initially removed, and the remaining articles were screened by titles/abstracts to determine the eligibility. Full-texts of the records that met the initial inclusion criteria were subsequently screened against the full study eligibility criteria.

The current meta-analysis included randomized controlled trials with exercise training vs. non-exercise (control) groups involving women, who were post-menopausal. Further inclusion criteria were as follows: English language, peer-reviewed; provided measures of main outcomes at baseline and also at post-intervention, and intervention duration ≥ 4 weeks. Participants included post-menopausal women with and without co-morbidities and regardless of age. For training, any type of exercise, including aerobic, resistance, interval, and combined training were included. In addition, other type of exercises, such as yoga and Tai chi were included. For the main outcomes, CRF was measured using VO_2peak/max_ tests including either maximal or submaximal test protocols (19). Lower- and upper-body muscular strength, as well as handgrip strength were measured *via* 1-repetition maximums (1RM) up to 10 repetition maximums (10RM), peak torque during isometric dynamometry, peak torque during isokinetic dynamometry, and handgrip strength. Lower-body muscular strength was assessed *via* leg press, and for upper-body, chest press was used. However, when these tests were not available, leg extension was used for lower-body muscular strength, and shoulder press or arm extension for upper-body muscular strength (15). Exclusion criteria included non-original studies, non-English language and non-full text articles, and interventions with durations of less than 4 weeks.

### Data extraction and synthesis

The following data were extracted from each included article: study design and year of publication, sample size, participant characteristics (biological sex, health status, age, and body mass index (BMI)), intervention characteristics (exercise training type, intensity, frequency, and duration), outcome variables and assessment methodologies. In addition, pre- and post-intervention means and standard deviations (SDs) or mean changes and their SDs of outcomes were included for the meta-analysis. Where the means and SDs were not reported, other data such as standard errors, medians, ranges, and/or interquartile ranges, were extracted and converted to means and SDs (17, 20, 21). The Getdata Graph Digitizer software was used for extraction of data from figures when necessary. For studies that had multiple type of intervention arms, only data from the exercise vs. non-exercised groups were included. For studies with multiple exercise arms, all comparisons were included as separate arms and the sample size of the control groups were divided to avoid double counting. For studies that did not provide sufficient information for the meta-analysis, we contacted the corresponding author of the articles. Two reviewer authors (A H M and M H S) independently extracted the data, and any disagreements were resolved *via* discussion with another reviewer (M Kh).

### Quality assessment and sensitivity analysis

The Physiotherapy Evidence Database (PEDro) tool was used to assess the methodological quality of all included studies. The PEDro tool contains 11 items, two of which, i.e., blinding of participants and intervention providers, were removed due to the lack of relevance of these items for exercise intervention studies. Therefore, the study quality of included studies was assessed using nine items, and determined by two independent reviewers (A H M and M H S). A higher score indicates a higher quality study (Supplementary Material Table S1). For the main analyses, sensitivity analysis was performed by eliminating individual studies to ensure that results were not significantly affected any individual study alone.

### Statistical analysis

Standardized mean differences (SMD), weighted mean differences (WMD), and 95% confidence intervals (CIs) were calculated using random effects models for comparing the effects of exercise training against non-exercise control groups on the main outcomes (i.e., CRF, lower- and upper-body muscular strength, and handgrip strength). Based on study aims, and to determine potential sources of heterogeneity, several subgroup analyses were conducted. Where there were more than 3 interventions for each subgroup, the following subgroup analyses were performed: middle-aged: < 65yrs and older adults: ≥ 65yrs; non-obese: BMI < 30 kg/m^2^ and obese: BMI ≥ 30 kg/m^2^; aerobic, resistance, combined, interval training; and intervention duration as medium-term: ≤ 16 weeks and long-term: > 16 weeks. Interpretation of effect sizes was conducted using Cochrane guidelines as follows: 0.20–0.49, 0.5–0.79, and >0.80 indicating small, medium, and large effect sizes, respectively. In addition, *I*^2^ statistics were used to evaluate heterogeneity and *I*^2^ statistics were interpreted as 25%, 50%, and 70% indicating low, moderate, and high heterogeneity, respectively. Visual interpretation of funnel plots and Egger's tests were used to assess the publication bias with *p* < 0.10 indicating significant publication bias. Where publication bias was detected using visual interpretation of funnel plots, the trim and fill method was used to correct the potential publication bias (22). All analyses were conducted using comprehensive meta-analysis software (CMA; Biostat, Englewood, NJ, Version 2).

## Results

### Description of included studies

**Figure 1:**
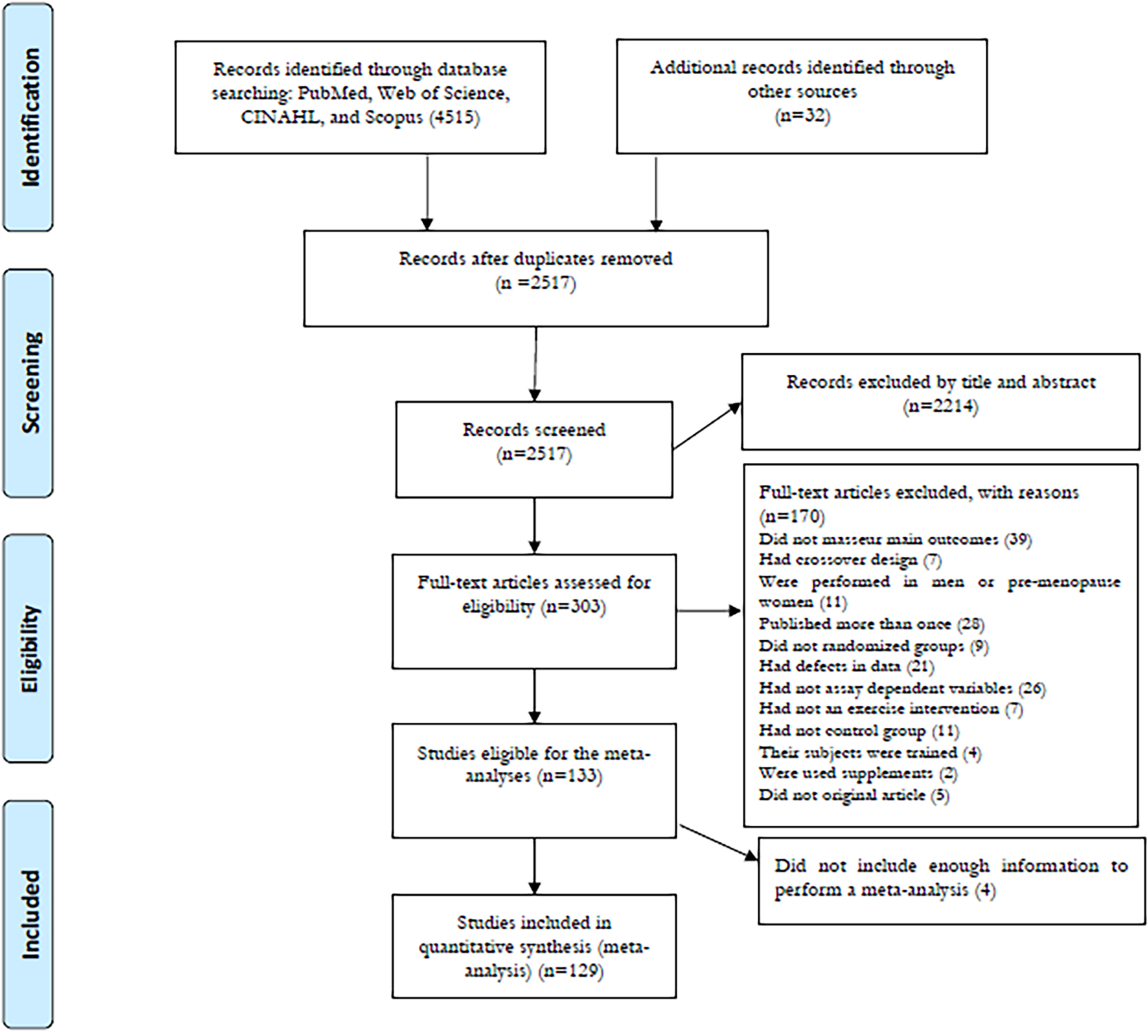
Flow diagram of systematic literature search.

The search strategy retrieved a total of 4,547 records from all databases, of which 2,517 articles were retained after removing the duplicates. Following screening of the titles and abstracts, 2,214 articles were excluded with 303 articles remaining for the full-text assessment according to the inclusion and exclusion criteria. Following the full-text screening, 174 articles were excluded for reasons provided in Figure 1. Finally, 129 studies were included in the meta-analysis, all randomized trials with parallel arms. The detailed screening process in each step according to the PRISMA guidelines is presented in Figure 1.

### Characteristics of participants and exercise training

The characteristics of participant and exercise training in each article are provided in Supplementary Material Table 1. Briefly, a total of 7,141 women who were post-menopausal were included in the meta-analysis. The age of women is ranging from ∼53 to 90 years, and their BMI is 22 to 35 kg/m^2^. All included studies recruited post-menopausal women, who were healthy or exhibited comorbidities such as obesity, chronic heart failure, nonalcoholic fatty liver disease, sarcopenia, hypertension, osteoarthritis, previous breast cancer, osteoporosis and vertebral fracture, fibromyalgia, or were community-dwelling with cognitive impairment. This approach allowed us to increase the potential generalizability of the results.

Most included studies adopted aerobic or resistance-based training alone, or in combination. Other studies used Tac chi, yoga, Pilates, and water-based training. Intervention durations was ranged from 6 weeks to 18 months, with exercise sessions ranging from 1 to 7 sessions per week. Most of the included studies used supervised exercise sessions and others used both supervised and unsupervised sessions in combination. However, several studies did not clearly report whether sessions were supervised or not. In addition, overall quality scores of included studies were summarized in Supplementary Material Table S1 which ranged from four to eight out of maximum of nine.

### Exercise training promotes CRF in post-menopausal women

**Figure 2:**
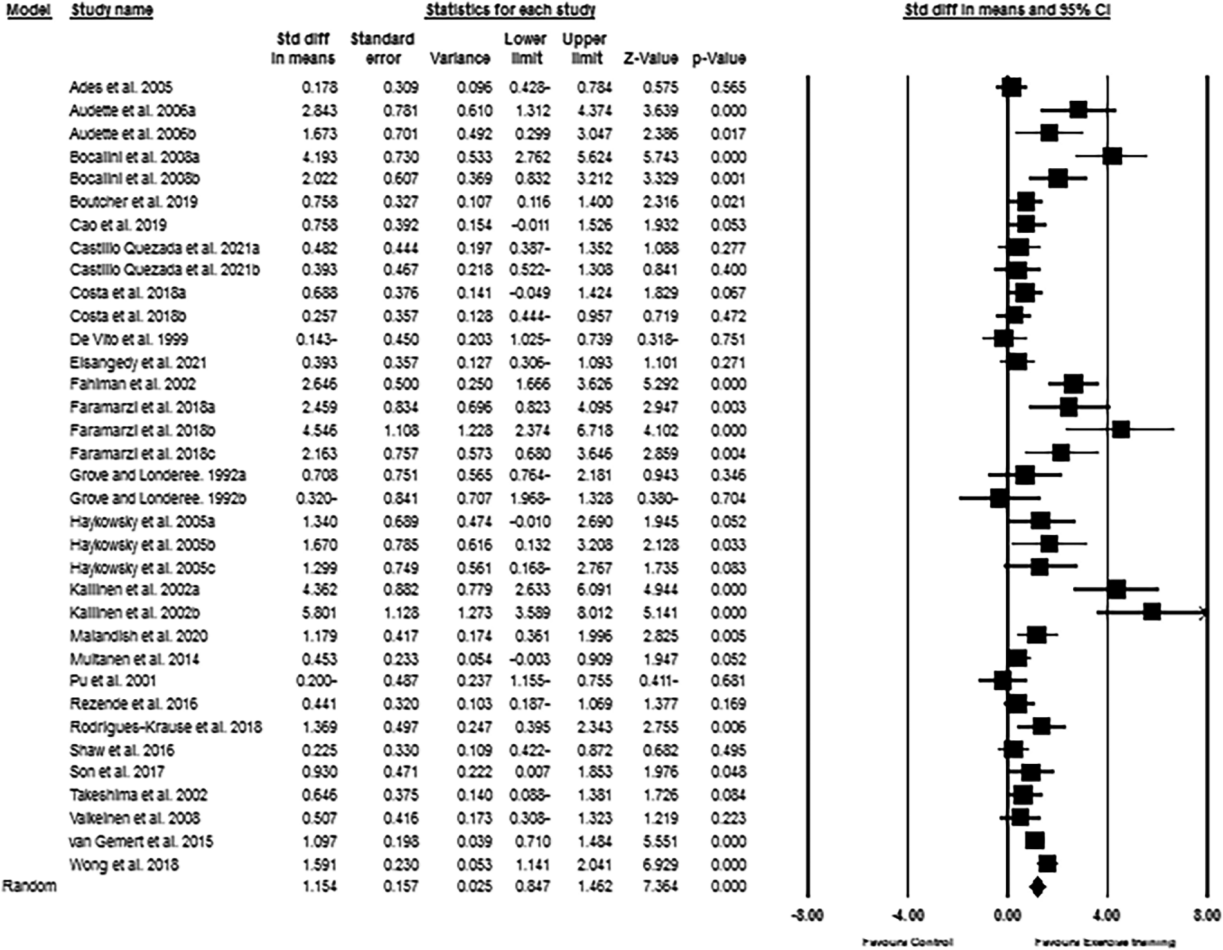
Forest plot of the effects of exercise training versus control on CRF. Data are reported as SMD (95% confidence limits). SMD: standardized mean difference.

Based on the results from 35 intervention arms from 25 studies, exercise training increased CRF (SMD: 1.15; 95% CI: 0.87, 1.42; *p* = 0.001) in post-menopausal women (Figure 2). There was a significant heterogeneity among the included studies (*I*^2 ^= 76.53%; *p* = 0.001). The Egger's test (*p* = 0.005) suggested publication bias, but visual interpretation of funnel plots did not indicate probable bias. In addition, sensitivity analysis by omitting individual studies did not change the direction or significance of the intervention effect. Results from age subgroup analyses revealed a significant increase of CRF in middle-aged participants (SMD: 1.06, *p* = 0.001) and older adults (SMD: 1.24, *p* = 0.001). For the exercise type, significant increases of CRF was found with aerobic training (SMD: 1.21, *p* = 0.001), resistance training (SMD: 1.26, *p* = 0.007), combined training (SMD: 1.47, *p* = 0.001), and water-based training (SMD: 0.83, *p* = 0.01). For the duration of intervention, increased CRF was found with medium-term interventions (SMD: 1.17, *p* = 0.001) and long-term interventions (SMD: 1.12, *p* = 0.001) (Supplementary Material Table S2).

### Exercise training improves lower-body muscular strength in post-menopausal women

**Figure 3:**
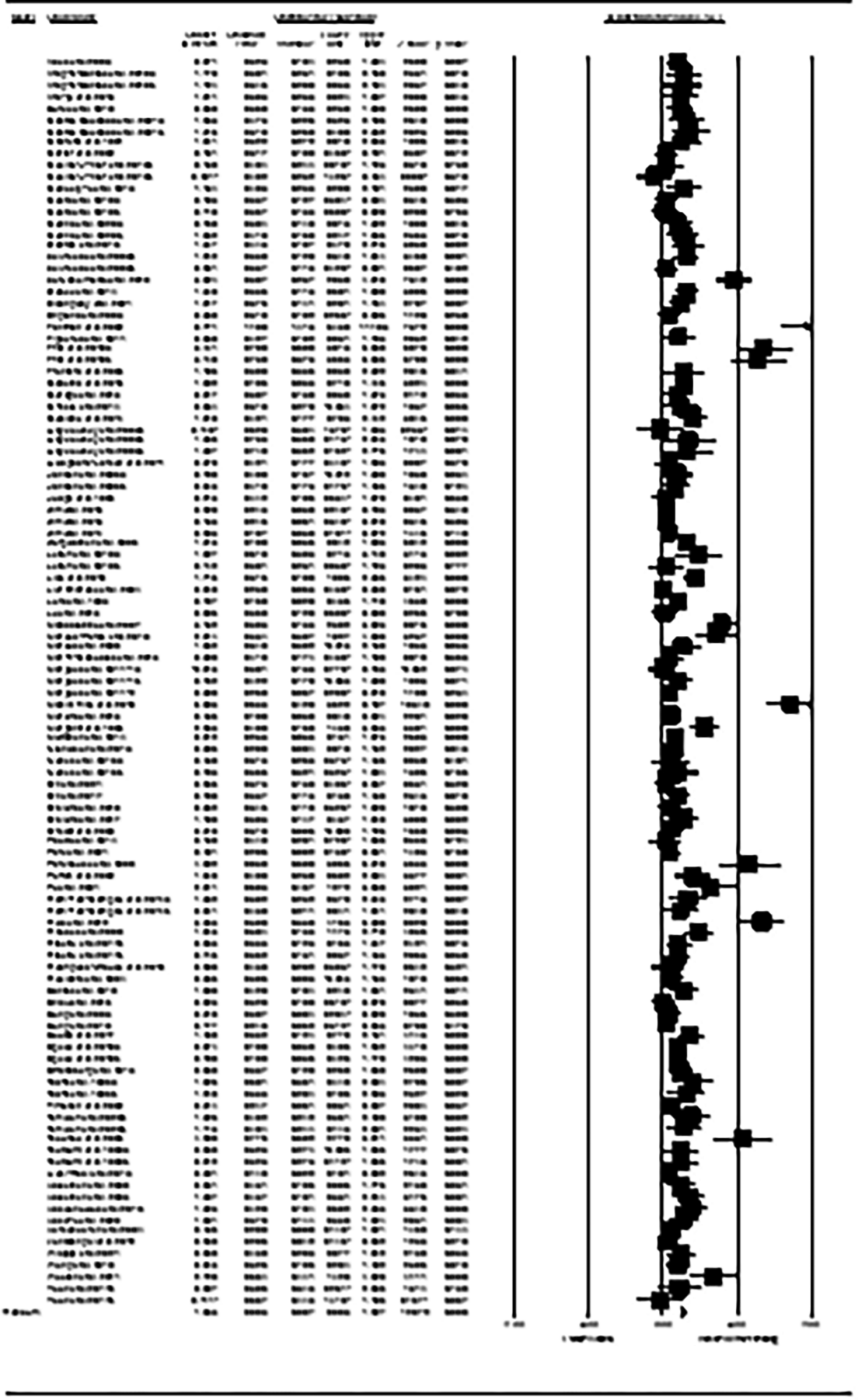
Forest plot of the effects of exercise training versus control on lower-body muscular strength. Data are reported as SMD (95% confidence limits). SMD: standardized mean difference.

A total of 109 intervention arms from 90 studies examined the exercise effect on lower-body muscular strength in post-menopausal women. Meta-analysis results showed that exercise training significantly increased the lower-body muscular strength in post-menopausal women (SMD: 1.06; 95% CI: 0.90, 1.22; *p* = 0.001) (Figure 3). We further noticed a significant heterogeneity among the included studies (*I*^2 ^= 82.08%; *p* = 0.001). The Egger's test (*p* = -.001) and visual interpretation of funnel plots both suggested publication bias. The trim and fill method identified 28 missing studies from the right side of the plots. When accounting for these missing studies, the overall change was 1.43 (95% CI: 1.25, 1.61). In addition, sensitivity analysis by omitting individual studies, did not change the direction or significance of the effect. Subgroup analyses revealed a significant increase of lower-body muscular strength in middle-aged participants (SMD: 0.98, *p* = 0.001) and older adults (SMD: 1.03, *p* = 0.001). Significant increases of lower-body muscular strength was also occurred with all exercise types, including aerobic (SMD: 0.49, *p* = 0.002), resistance (SMD: 1.28, *p* = 0.001), combined (SMD: 0.61, *p* = 0.001), and water-based (SMD: 0.96, *p* = 0.02) trainings. Furthermore, both medium-term interventions (SMD: 1.30, *p* = 0.001) and long-term interventions (SMD: 0.82, *p* = 0.001) significantly improved the lower-body muscular strength in post-menopausal women (Supplementary Material Table S2).

### Resistance training increases upper-body muscular strength in post-menopausal women

**Figure 4:**
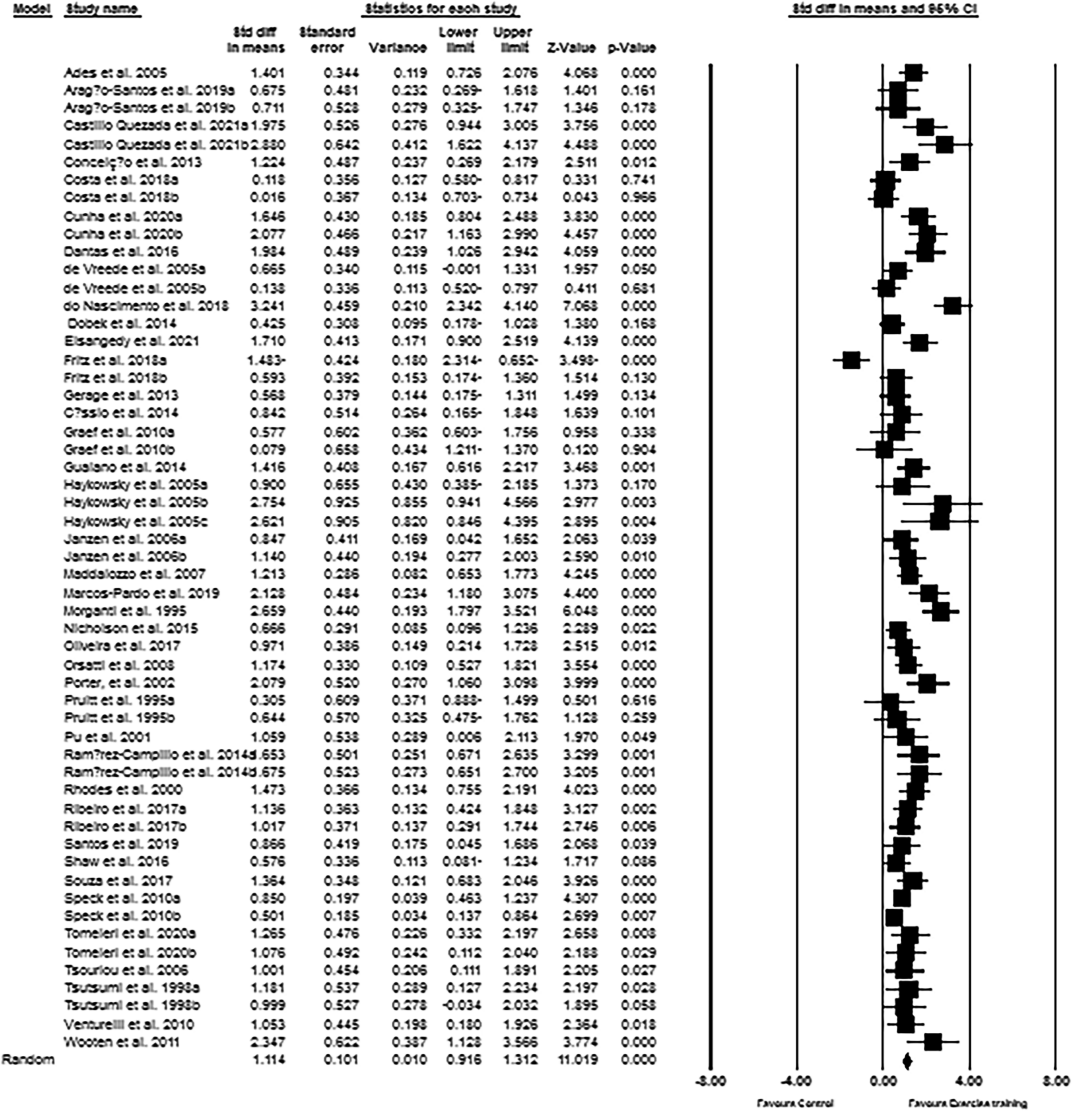
Forest plot of the effects of exercise training versus control on upper-body muscular strength. Data are reported as SMD (95% confidence limits). SMD: standardized mean difference.

The upper-body muscular strength data from 55 intervention arms were used in the meta-analysis. The results revealed that exercise training increased upper-body muscular strength (SMD: 1.11; 95% CI: 0.91, 1.31; *p* = 0.001) in post-menopausal women (Figure 4). There was a significant heterogeneity among the included studies (*I*^2 ^= 68.84%; *p* = 0.001). The assessment results from both Egger's test (*p* = 0.004) and visual interpretation of funnel plots suggested publication bias. The trim and fill method identified 14 missing studies from the right side of the plots. When accounting for these missing studies, the overall change was 0.80 (95% CI: 0.58, 1.01). Subgroup analyses revealed that upper-body muscular strength was significantly increased in both middle-aged (SMD: 1.02, *p* = 0.001) and older adults (SMD: 1.15, *p* = 0.001). For the exercise type, we found resistance training significantly increased upper-body muscular strength in women (SMD: 1.20, *p* = 0.007), and the effect size is relatively bigger. To be specific, both medium-term interventions (SMD: 1.13, *p* = 0.001) as well as long-term interventions (SMD: 1.08, *p* = 0.001) of exercise training improved upper-body muscular strength in post-menopausal women (Supplementary Material Table S2).

### Resistance and combined trainings increase handgrip strength of post-menopausal women

**Figure 5:**
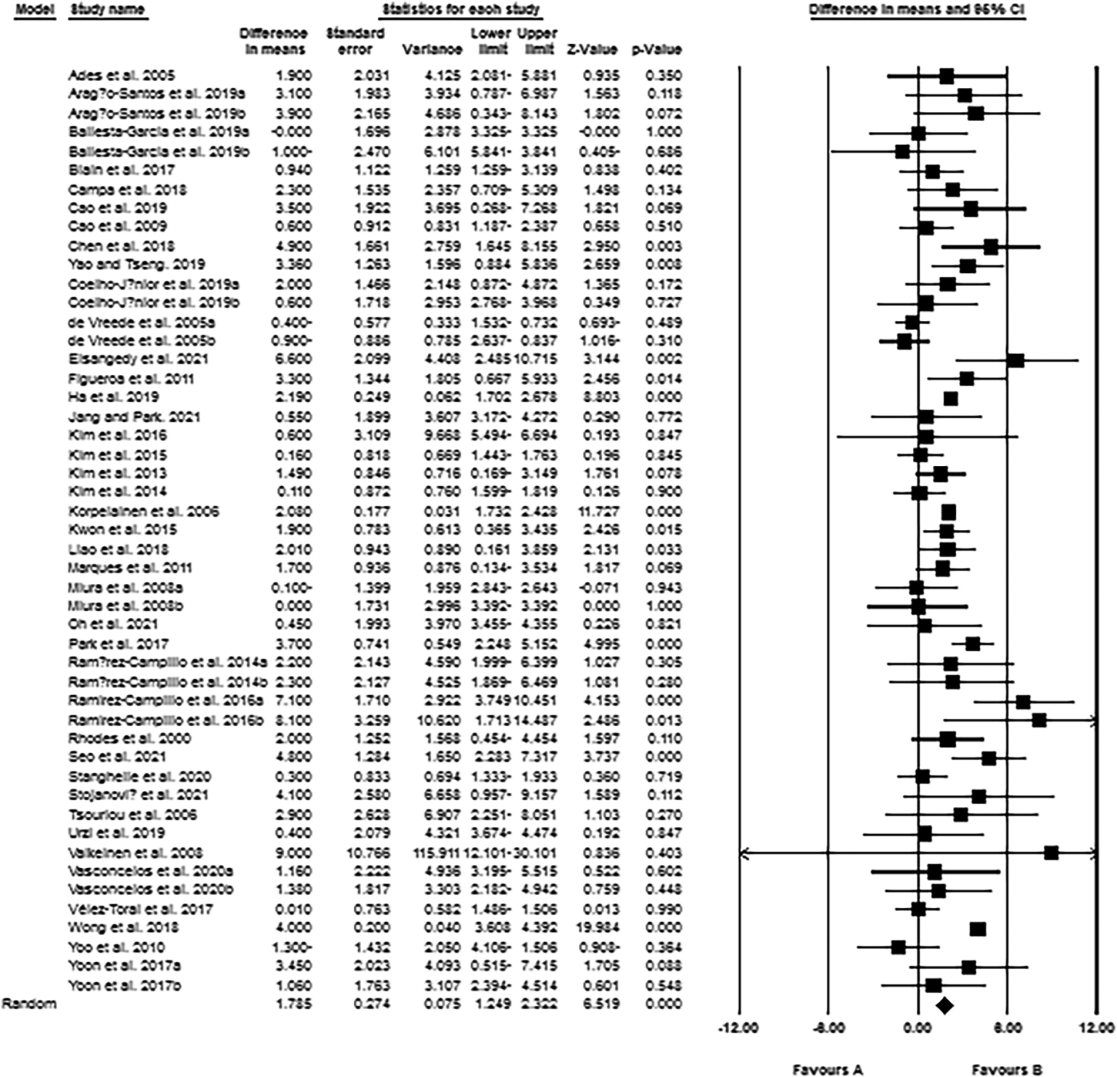
Forest plot of the effects of exercise training versus control on handgrip strength. Data are reported as WMD (95% confidence limits). WMD: weighted mean difference.

The meta-analysis results from 49 intervention arms indicated that exercise training effectively increased handgrip strength (WMD: 1.78 kg; 95% CI: 1.24, 2.32; *p* = 0.001) of post-menopausal women (Figure 5). We further noticed a significant heterogeneity among the included studies (*I*^2 ^= 74.91%; *p* = 0.001). Visual interpretation of the Egger's test (*p* = 0.09) and funnel plots both suggested publication bias of the studies. We identified 2 missing studies from the left side of the plots using trim and fill method. When accounting for these missing studies, the overall change was 1.74 kg (95% CI: 1.20, 2.28). In addition, sensitivity analysis performed by omitting individual studies, did not change the direction or significance of the exercise effect. Subgroup analyses revealed a significant increase of handgrip strength in middle-aged (WMD: 2.49 kg, *p* = 0.006), as well as in older adults (WMD: 1.69 kg, *p* = 0.001). Both resistance training (WMD: 1.72 kg, *p* = 0.001) and combined training (WMD: 1.82 kg, *p* = 0.001) increased the handgrip strength of women, but not aerobic training. For the duration, both medium-term interventions (WMD: 1.83 kg, *p* = 0.001), and long-term interventions (WMD: 1.82 kg, *p* = 0.001) increased the handgrip strength in post-menopausal women (Supplementary Material Table S2).

## Discussion

For the first time, our systematic review and meta-analysis provides a comprehensive analysis of a large body of evidence on the effects of exercise training on CRF and muscular strength outcomes in post-menopausal women. The results demonstrated that exercise training is effective in increasing the CRF, and lower- and upper-body muscular strength, as well as handgrip strength of women. Subgroup analyses results revealed that these beneficial effects of exercise training were not limited to age of participants (middle-aged or older) or duration of intervention (medium-term or long-term). For the type of exercise, aerobic, resistance, and combined exercise trainings efficiently increased CRF and lower-body muscular strength, whereas increased upper-body muscular strength was noticed only with resistance exercise training. Besides, both resistance and combined exercise trainings significantly increased the handgrip strength of post-menopausal women.

It has been documented that poor CRF is a risk factor for non-communicable chronic diseases, such CVDs and other metabolic disorders, which then collectively increase the mortality risk (23–28). In addition, age-related decrease of CRF has been reported as an important predictor of hospitalization, morbidity, and mortality in older adults (29, 30). Evidence from a recent study suggests that CRF declines with advancing age, and this decline further accelerates in women during the menopausal transition (8). Cardiometabolic diseases are thought to be promoted by poor CRF through several possible physiological mechanisms, including effects on insulin function and secretion, increased insulin resistance, poor lipid profiles, increased blood pressure, inflammation, and chronic oxidative stress (1). Multiple events, including decreased maximal heart rate, maximal stroke volume, maximal cardiac output, blood volume, muscular mass, arteriovenous oxygen difference, and muscular blood flow are involved in reducing the CRF (31–33), and all these episodes occur with decreased physical activity (33). Therefore, effective treatment strategies are crucial to maintain the CRF during aging, especially in post-menopausal women.

Evidence from our meta-analysis revealed that exercise training is an effective intervention to promote CRF in post-menopausal women. These findings are in agreement with previous reviews, which summarizes the beneficial effects exercise training in patients with type 2 diabetes (34), stroke (35), and older adults (15, 36, 37). However, to the best of our knowledge, this is the first comprehensive meta-analysis to report the cardioprotective effects of exercise in post-menopausal women with a large sample size. Since poor CRF is considered an important predictor of all-cause mortality in women (38), improving CRF could alleviate the menopausal-associated symptoms (39). The results from our meta-analysis indicate clinically meaningful improvements in CRF following aerobic, resistance, or combined exercise training, with potential greater advantages of combined training. Exercise training has beneficial effects on CRF possibly *via* two main mechanisms, including central circulatory adaptations (mainly improving cardiac output and arteriovenous O_2_ differences) and peripheral adaptations (primarily improving skeletal muscle mitochondrial function and increasing capillaries) (40). Our subgroup analyses results further emphasize the clinical importance of “exercise intervention type” on improving the CRF in women. The American College of Sports Medicine (ACSM) recommended combination of aerobic and resistance exercise programs for maintaining of cardiorespiratory and musculoskeletal fitness in healthy adults (41). Nevertheless, our findings revealed that any mode of exercise, including aerobic, resistance, or combined training is effective in improving the CRF in post-menopausal women. These findings indicate that post-menopausal women can get beneficial effects with either type of exercise intervention. Some previous meta-analyses also claimed the positive effects of aerobic (10), high-intensity interval (42), resistance (43), and combined (15, 44, 45) training on CRF in adults.

Loss of muscular strength is associated with disability and all-cause mortality in several clinical populations (46), and is also important for post-menopausal women as women experience dramatic loss of muscular strength after menopause (47, 48). Muscular strength, is therefore a modifiable risk factor that could be intervened among women who are post-menopausal and/or as women enter into the menopausal transition. Previous meta-analyses have confirmed the effectiveness of exercise training, especially resistance training on muscular strength gains (14, 15, 49). However, to the best our knowledge, no meta-analysis has been confirmed the exercise effect in post-menopausal women. Results from our meta-analysis indicated that exercise training effectively increased lower- and upper-body muscular strength, as well as handgrip strength in post-menopausal women. These results were consistent with previous meta-analyses in adults, older adults, and very old adults, as well as in patients with type 2 diabetes (13, 15, 50–52). The current meta-analyses also evaluated the specific type of exercise, and showed that resistance training was effective in improving all three strength outcomes including lower- and upper-body muscular strength, and handgrip strength among women. On the other hand, aerobic training was also effective in improving the lower-body muscular strength, and not upper-body muscular strength. Therefore, a combination of aerobic and resistance training may be optimal in women to maintain their physical function and daily living activities during or after menopause. Resistance training has been recommended as an effective and safe intervention for all ages, to prevent muscular strength loss according to a position statement by the ACSM (41, 53). Resistance training-induced increased muscular strength depends on exercise intensity and volume, but, regardless of those variables, resistance exercise training can improved strength through neural, muscular, and metabolic adaptations. Therefore, it is not surprising that resistance exercise training is effective in increasing the muscular strength in post-menopausal women. In particular, despite lower baseline muscular strength in women when compared with men, meta-analytic evidence suggests that older women exhibited greater relative increases compared to older men in lower-body strength, with no sex differences in changes of relative upper-body strength (54). Although specificity of training suggests that aerobic training is mainly used to improve cardiorespiratory fitness and reduce fat mass, these meta-analytic results indicated that aerobic and combined training are also effective in increasing lower-body muscular strength, though the effect sizes were not as large as the effects of resistance training alone. There are some studies stating that aerobic based training may have antagonistic effects on increasing muscular mass and strength (55–58); however, these previous results may not be generalizable in post-menopausal women or older adults (15, 59, 60), and lower baseline muscular strength may allow for achievement of greater adaptations (60).

Furthermore, subgroup analyses showed that beneficial adaptation in CRF and muscular strength occurred regardless of intervention durations and ages of participants. These are clinically important findings and show that the beneficial effects of exercise can be obtained at any age and with both medium- and long-term intervention durations in post-menopausal women. However, there are few limitations that should be considered. For both CRF and muscular strength we included studies regardless of the methods and units of measurement of outcomes, which did not allow us to use WMD for calculation of effect sizes. Subsequently, we were not able to report units of change that were easily interpretable for assessment of clinical meaning of the effects. Finally, a significant heterogeneity indicated for all results, which may be due to the differences in ages and health statuses of the participants; components of the exercise protocols, such as intensity, duration, or frequency; as well as measurement methods of the reported outcomes.

## Conclusion

Our systematic review and meta-analysis provides a large-body of evidence that exercise training is effective in improving the CRF and muscular strength in post-menopausal women. To be specific, increased CRF was noticed irrespective of exercise type, exercise duration and age of post-menopausal women. Both aerobic and resistance training alone or in combination, are effective in improving the lower-body muscular strength, but resistance training was the only exercise type that improved upper-body muscular strength in women. The greater beneficial effects of exercise (either type or any duration) on CRF indicating the potential clinical importance of exercise intervention to treat or prevent the CVDs in post-menopausal women (middle-aged or elderly).

## Perspectives

Based on our findings, it is suggested that any type of exercise training (aerobic, resistance or combination of both) at moderate-intensity with a duration of 8-week or more would be beneficial in improving the cardiorespiratory fitness among post-menopausal women. For the improvement of whole-body muscular strength, moderate-intensity resistance training with a duration of more than 8-week, would be optimum. However, practicing of aerobic exercise or combination of aerobic and resistance exercise also improve lower-body muscular strength. For the frequency and time of exercise, at least 3 sessions and a total of 150 min of physical activity per week would be practically advisable to post-menopausal women to achieve the adequate beneficial effects.

## Future studies

In this meta-analysis, we primarily focused and examined the effects of exercise training in women with post-menopause stage. We haven't included studies involving women with menopause, and therefore, we couldn’t compare the differences between post-menopausal and menopausal women in response to exercise training. On the other hand, a recent meta-analysis stated that there is no biological sex differences in relative muscle strength gain in response to resistance exercise training (54). However, the sex-specific response of other outcomes, like cardiorespiratory fitness, lower-body muscular strength and upper-body muscular strength in response to other types of exercises remains inconclusive. Therefore, there is a scope for further studies to examine and compare the exercise-mediated beneficial effects between menopausal and post-menopausal women, as well as between age-matched males and females on cardiorespiratory fitness and muscular strength.

## Data Availability

The original contributions presented in the study are included in the article/**Supplementary Material**, further inquiries can be directed to the corresponding authors.
