# Supplementary material for: Influence of exercise type and duration on cardiorespiratory fitness and muscular strength in post-menopausal women: a systematic review and meta-analysis
Source: Front Cardiovasc Med. 2023 May 9;10:1190187. doi: 10.3389/fcvm.2023.1190187 (PMC10204927; doi:10.3389/fcvm.2023.1190187)
Supplement: Supplementary file 1 [file Table1.docx]

| **Source, year** | **Participant characteristics** | | | | **Exercise training characteristics** | | | | | **Outcomes**  **(measurement methods)** |
| --- | --- | --- | --- | --- | --- | --- | --- | --- | --- | --- |
|  | **Sample size** | **Participant health status** | **Age [years]** | **BMI [kg/m^2^]** | **Exercise type** | **Description of exercise intervention** | **Supervised or unsupervised** | **Duration** | **Frequency**  **(d/week)** |  |
| Ades et al., 2005 [62] | 42 | disabled with coronary heart disease | EX:72.9±6.1  CON:71.5±4.8 | EX:28.7±4.2  CON:30.2±5.6 | Resistance | whole body exercises; 1-2 sets of 10 reps at 50-80% of 1RM | Supervised | 6 months | 3 | VO_2peak_ (Balke-gas analyzer), UBS, LBS (1RM), HG (dynamometer) |
| Aragão-Santos et al., 2019 [63] | 55 | healthy | EX_1_:65.6±5.4  EX_2_:65.6±5.1  CON:62.5±3.0 | EX_1_:29.0±4.9  EX_2_:28.5±5.5  CON:30.4±5.9 | EX_1_: Functional  EX_2_: Combined | EX_1_: 15-min agility, coordination, muscle power, speed and cardiorespiratory exercises at a rating of 6-7 BRPE + 25 min multi-joint exercises at a rating of 7-9 BRPE  EX_2_: 15-min aerobic exercises at a rating of 6-7 BRPE + 25-min whole body circuit exercises; 2 sets of 8-12 reps at a rating of 7-9 BRPE | ND | 12 weeks | 3 | UBS, LBS (1RM), HG (dynamometer) |
| Araújo et al., 2015 [64] | 18 | healthy | EX:53.0±3.0  CON:55.0±3.0 | EX:26.4±4.6  CON:24.9±2.6 | Water-based exercise | four sets, one with 30 reps, and three with 15 reps at a rating of 9-11 BRPE with 1 min recovery | Supervised | 8 weeks | 3 | LBS (1RM) |
| Audette et al., 2006 [65] | 27 | elderly | EX_1_:71.5±4.6  EX_2_:71.3±4.4  CON:73.5±5.7 | ND | EX_1_: Tai chi  EX_2_: Aerobic | EX_1_: 40-45 min of the 10-movement form  EX_2_: 40-min at 50-70% of HR_max_ | Supervised | 12 weeks | 3 | VO_2max_ (YMCA exercise test) |
| Baggen et al., 2019 [66] | 45 | elderly | EX:69.0±4.0  CON:69.0±4.0 | EX:26.8±4.7  CON:25.1±3.4 | Resistance | bench stepping exercises; 2 sets of 32 reps at 60% of 1RM | Supervised | 12 weeks | 3 | LBS (isokinetic dynamometer) |
| Ballesta-García et al., 2019 [67] | 54 | healthy | EX_1_:66.3±5.4  EX_2_:70.0±8.9  CON:67.4±5.7 | EX_1_:30.4±4.1  EX_2_:30.1±3.1  CON:31.2±4.9 | Resistance | EX_1_: whole body circuit exercises; at a rating of 16-18 BRPE  EX_2_: whole body circuit exercises; at a rating of 12-14 BRPE | Supervised | 18 weeks | 2 | HG (dynamometer) |
| Blain et al., 2017 [68] | 121 | physically deconditioned | EX:65.6±4.4  CON:65.8±4.2 | EX:24.3±3.5  CON:26.6±4.2 | Aerobic | 50-min at 40-80% of HR_max_ | Supervised & Unsupervised | 6 months | 3 | HG (dynamometer) |
| Bocalini et al., 2008 [69] | 72 | healthy | EX_1_:64.0±1.0  EX_2_:64.0±1.0  CON:63.0±1.0 | ND | EX_1_: Resistance  EX_2_: Aerobic | EX_1_: whole body endurance-type exercise; with 10-15 reps, at 70% of HR_max_  EX_2_: 45 min at 70% of HR_max_ | Supervised | 12 weeks | EX_1_: 3  EX_2_: 5 | VO_2max_ (Bruce) |
| Boutcher et al., 2019 [70] | 40 | overweight | EX:54.1±3.6  CON:53.3±3.4 | EX:28.3±3.7  CON:27.3±4.1 | HIIT | sixty reps of 8-s at near-maximal exertion with 12-s recovery at 80-85% of HR_max_ | Supervised | 8 weeks | 3 | VO_2max_ (submaximal exercise test; cycle ergometer) |
| Burke et al., 2010 [71] | 33 | osteoporosis | EX:72.8±3.6 CON:74.4±3.7 | EX:26.3±2.2  CON:25.4±2.3 | Resistance | 30 min lower body exercises; with 10-15 reps, weight (1-2 kg) was increased if participants were able to satisfactorily repeat exercises 15 times + 20 min balance exercises | Supervised | 8 weeks | 2 | LBS (isometric dynamometer) |
| Campa et al., 2018 [72] | 30 | healthy | EX:66.5±4.3  CON:65.6±5.2 | EX:28.8±4.6  CON:32.4±5.6 | Resistance | whole body suspension exercises; 4 sets of 12 reps | Supervised | 12 weeks | 2 | HG (dynamometer) |
| Cao et al., 2019 [73] | 30 | overweight and obese | EX:63.8±5.9  CON:64.0±4.6 | EX:28.0±2.9  CON:26.4±1.4 | Aerobic | 20-40 min at the individualized FAT max HR | Supervised | 12 weeks | 3 | VO_2max_ (gas analyzer), HG (dynamometer) |
| Cao et al., 2009 [74] | 126 | healthy | EX:63.2±4.5  CON:66.5±4.3 | EX:23.7±2.2  CON:22.5±2.3 | Combined | performed aerobic exercise, antigravity exercise, circuit training, and resistance exercises; lower body resistance exercises; 1-2 sets of 20-30 reps | Supervised & Unsupervised | 12 months | 3-4 | HG (dynamometer) |
| Carrasco-Poyatos et al., 2019 [75] | 60 | healthy | EX_1_:67.5±3.9  EX_2_:73.4±4.8  CON:65.9±4.5 | EX_1_:32.3±5.2  EX_2_:31.1±4.8  CON:30.5±6.4 | EX_1_: Pilates  EX_2_: Resistance | Pilates and resistance training exercise programs were focused on the spine, hip and girdle regions, stimulating the muscles in a dynamic and static way and exercising the arms and legs; with 4-12 reps at a rating of 4-9 on OMNI scale | Supervised | 18 weeks | 2 | LBS (isokinetic dynamometer) |
| Carter et al., 2002 [76] | 93 | osteoporosis | EX:69.6±3.0  CON:69.0±3.5 | ND | Resistance | Upper body strengthening and stretching exercises with elastic bands and small free weights (1-2 kg) for 40 min | Supervised | 20 weeks | 2 | LBS (using a strap assembly) |
| Castillo Quezada et al., 2021 [77] | 63 | healthy | EX_1_:69.4±6.5  EX_2_:69.4±6.5  CON:69.4±6.5 | ND | EX_1_: Aerobic  EX_2_: Resistance | EX_1_: 45-min at 50-70% of HR_max_  EX_2_: whole body exercises; 2-4 sets of 10-15 reps at 50-60% of 1RM | Supervised | 12 weeks | 3 | VO_2max_ (6 min walking test), UBS, LBS (1RM) |
| Charette et al., 1991 [78] | 27 | healthy | EX:69.8±4.3  CON:67.7±4.2 | ND | Resistance | resistance exercises; 3-6 sets of 6 reps at 65-75% of 1RM | Supervised | 12 weeks | 3 | LBS (1RM) |
| Chen et al., 2018 [79] | 33 | sarcopenia | EX:66.7±5.3  CON:68.3±2.8 | ND | Resistance | whole body kettlebell training; 3 sets of 8-12 reps at 60-70% of 1RM | Supervised | 8 weeks | 2 | HG (dynamometer) |
| Yao and Tseng. 2019 [80] | 31 | low physical activity levels | EX:76.4±6.1  CON:78.7±6.2 | EX:24.8±3.1  CON:25.7±3.8 | Chair yoga | 80-min chair yoga (sitting position strength training, stretching, standing position balance training) | Supervised | 12 weeks | 2 | HG (dynamometer) |
| Coelho-Júnior et al., 2019 [81] | 45 | healthy | EX_1_:67.0±6.2  EX_2_:66.7±5.1  CON:66.7±4.6 | EX_1_:30.2±4.1  EX_2_:27.8±6.2  CON:27.0±7.7 | Resistance | EX_1_: non-periodized resistance exercises; 1-3 sets of 8-15 reps at a rating of 5-10 BRPE  EX_2_: daily undulating periodization resistance exercises; 1-3 sets of 8-15 reps at a rating of 5-10 BRPE | Supervised | 22 weeks | 2 | HG, LBS (isometric dynamometer) |
| Conceição et al., 2013 [82] | 20 | healthy | EX:53.4±3.9  CON:53.0±5.7 | EX:26.2±3.3  CON:25.3±1.8 | Resistance | whole body exercises; 3 sets of 8-10 reps | Supervised | 16 weeks | 3 | UBS, LBS (1RM) |
| Costa et al., 2018 [83] | 69 | healthy | EX_1_:66.8±5.2  EX_2_:66.8±4.5  CON:64.6±5.5 | EX_1_:28.8±4.0  EX_2_:29.9±5.7  CON:30.9±6.6 | Water-based exercise | EX_1_: 30-min water-based aerobic exercise at 80-100% of HR_AT_ (anaerobic threshold)  EX_2_: whole body water-based resistance exercises; 4-8 sets of 10-20 seconds | Supervised | 10 weeks | 2 | VO_2peak_ (Bruce & gas analyzer),  UBS, LBS (1RM) |
| Cunha et al., 2020 [84] | 69 | healthy | EX_1_:70.1±5.9  EX_2_:68.6±4.4  CON:68.0±4.4 | EX_1_:27.8±5.0  EX_2_:26.7±4.8  CON:26.4±4.5 | Resistance | EX_1_: whole body single-set exercises; 1 set of 10-15 reps  EX_2_: whole body multiple-sets exercises; 3 sets of 10-15 reps | Supervised | 12 weeks | 3 | UBS, LBS (1RM) |
| Dantas et al., 2016 [85] | 25 | hypertensive | EX:64.7±4.7  CON:67.7±5.6 | EX:28.6±3.2  CON:27.7±3.7 | Resistance | whole body exercises; 10 reps at a rating 5-7 OMNI scale | Supervised | 10 weeks | 2-3 | UBS, LBS (1RM) |
| De Vito et al., 1999 [86] | 22 | healthy | EX:63.0±3.1  CON:63.5±3.3 | ND | Aerobic | 20-25 min walking linked with upper- and lower-limb exercises at 60% of HRR | Supervised | 12 weeks | 3 | VO_2max_ (cycling test-gas analyzer) |
| de Vreede et al., 2005 [87] | 98 | healthy | EX_1_:74.7±3.5  EX_2_:74.8±4.0  CON:73.0±3.2 | ND | EX_1_: Functional EX_2_: Resistance | EX_1_: 40 min, at a rating of 7-8 BRPE  EX_2_: whole body dumbbell and elastic band exercises; 3 sets of 10 reps at a rating of 7-8 BRPE | Supervised | 12 weeks | 3 | UBS, LBS, HG (isometric dynamometer) |
| do Nascimento et al., 2018 [88] | 44 | healthy | EX:66.3±4.8  CON:66.4±4.0 | EX:24.9±3.3  CON:26.2±2.6 | Resistance | whole body exercises; 2 sets of 10 reps until moderate fatigue in each exercise or when it began to get difficult | Supervised | 12 weeks | 3 | UBS, LBS (1RM) |
| Dobek et al., 2014 [89] | 67 | breast cancer survivors | EX:64.2±6.0  CON:63.8±7.3 | EX:28.9±5.5  CON:27.9±4.5 | Resistance + Impact training | whole body dumbbell and barbells and weighted vests exercises; 2 sets of 8-12 reps | Supervised & Unsupervised | 12 months | 3 | UBS, LBS (1RM) |
| Elsangedy et al., 2021 [90] | 32 | healthy | EX:65.7±3.3  CON:66.3±2.8 | EX:26.5±3.6  CON:25.3±3.3 | Resistance | self-selected resistance exercises: 1-3 sets of 5-10 reps at low to high load | Supervised | 12 weeks | 3 | UBS, LBS (1RM), HG (dynamometer), VO_2max_ (Bruce-gas analyzer) |
| Englund et al., 2005 [91] | 48 | healthy | EX:72.8±3.6  CON:73.2±4.9 | EX:25.2±2.7  CON:26.1±3.2 | Combined | 10-min steps combinations with coordinated arm movements + lower and core resistance exercises; 2 sets of 8-12 reps + static balance and coordination | Supervised | 12 months | 2 | LBS (tensiometer) |
| Fahlman et al., 2002 [92] | 45 | healthy | EX_1_:76.0±5.0  EX_2_:73.0±3.0  CON:74.0±5.0 | EX_1_:26.0±5.0  EX_2_:26.0±5.0  CON:26.0±2.0 | EX_1_: Aerobic  EX_2_: Resistance | EX_1_: 20-50 min at 70% of HRR  EX_2_: lower body exercises; 1-3 sets of 8 reps at 8RM | Supervised | 10 weeks | 3 | VO_2max_ (1-mile walk test), LBS (1RM) |
| Faramarzi et al., 2018 [93] | 40 | overweight | EX_1_:60.3±0.8  EX_2_:60.3±0.8  EX_3_:60.3±0.8  CON:60.3±0.8 | EX_1_:29.9±1.2  EX_2_:29.2±1.7  EX_3_:27.6±0.9  CON:31.7±0.9 | EX_1_: Aerobic - resistance  EX_2_: Resistance – aerobic  EX_3_: Alternative combined | A: 16-30 min at 60-88% of HR_max_  R: whole body exercises; 2-3 sets of 8-18 reps at 40-75% of 1RM | Supervised | 10 weeks | 3 | VO_2max_ (Bruce) |
| Figueroa et al., 2011 [94] | 24 | healthy | EX:54.0±6.9  CON: 54.0±3.5 | EX:24.2±2.4  CON:23.1±2.4 | Combined | A: 20-min at 60% of HR_max_  R: whole body circuit exercises; 1 set of 12 reps at 60% of 1RM | Supervised | 12 weeks | 3 | LBS (1RM), HG (dynamometer) |
| Fritz et al., 2018 [95] | 75 | overweight | EX_1_:69.2±1.1  EX_2_:70.4±1.0  CON:67.2±1.1 | EX_1_:27.4±1.0  EX_2_:29.0±0.9  CON:26.5±0.8 | Resistance | EX_1_: whole body traditional elastic band exercises; 3-4 sets of 10 reps at a rating of 7-9 on OMNI scale  EX_2_: whole body elastic tubes with handles exercises; 3-4 sets of 10 reps at a rating of 7-9 on OMNI scale | Supervised | 8 weeks | 2 | UBS, LBS (1RM) |
| Frontera et al., 2003 [96] | 14 | healthy | EX:73.7±3.4  CON:74.3±3.8 | EX:23.7±3.6  CON:23.2±1.6 | Resistance | lower body exercises; 4 sets of 8 reps at 65-80% of 1RM | ND | 12 weeks | 3 | LBS (1RM) |
| Gadelha et al., 2016 [97] | 133 | healthy | EX:66.8±5.4  CON:67.3±5.0 | EX:27.1±4.0  CON:29.1±5.1 | Resistance | whole body exercises; 3 sets of 8-12 reps at 60-80% of 1RM | Supervised | 24 weeks | 3 | LBS (isokinetic dynamometer) |
| Gerage et al., 2013 [98] | 31 | healthy | EX:65.5±5.0  CON:66.2±4.1 | EX:23.9±2.9  CON:25.1±3.4 | Resistance | whole body exercises; 2 sets of 10-15 reps until moderate fatigue in each exercise or stopped when it began to be diﬃcult | Supervised | 12 weeks | 3 | UBS, LBS (1RM) |
| Cássio et al., 2014 [99] | 17 | hypertensive | EX:65.6±5.0  CON:66.1±3.8 | EX:27.1±4.2  CON:27.8±1.9 | Resistance | whole body exercises; 2 sets of 15 reps at 40% of 1RM | ND | 12 weeks | 3 | UBS, LBS (1RM) |
| Graef et al., 2010 [100] | 27 | healthy | EX_1_:68.4±6.7  EX_2_:64.1±3.5  CON:67.6±4.7 | EX_1_:27.1±2.3  EX_2_:26.0±3.5  CON:25.8±3.6 | Water-based exercise | EX_1_: EX_2 +_ whole body water-based resistance exercises; 4-5 sets of 8-15 reps  EX_2_: 50-min water-based aerobic exercises included stationary running, hamstring curl, front kick, jumping jacks, cross country ski, twist; at a rating of 11-13 BRPE | ND | 12 weeks | 2 | UBS (1RM) |
| Grove and Londeree. 1992 [101] | 15 | healthy | EX_1_:54.0±1.9  EX_2_:56.6±4.3  CON:56.0±4.5 | ND | EX_1_: High impact exercise  EX_2_: Low impact exercise | Exercises using a force plate  EX_1_: greater than or equal to two times the body weight, jumping jack (3.29 x body weight), running-in-place (2.47 x body weight), and knee-to-elbow with jump (2.79 x body weight)  EX2: less than 1.5 times the body weight, slow walk (1.19 x body weight), fast walk (1.49 x body weight), heel jack without a jump (1.34 x body weight), and the Charleston (1.32 x body weight) | Supervised | 12 months | 3 | VO_2max_ (Balke treadmill test) |
| Gualano et al., 2014 [102] | 30 | healthy | EX:63.6±3.6  CON:66.3±6.0 | EX:28.2±3.6  CON:26.8±5.5 | Resistance | 3 sets of 8-12 reps, except during the first week, which was at reduced volume of 2 sets of 15-20 reps | Supervised | 24 weeks | 2 | UBS and LBS (1RM) |
| Ha et al., 2019 [103] | 19 | healthy | EX:74.1±4.2  CON:76.0±5.5 | EX:23.9±1.8  CON:29.1±12.5 | Water-based exercise | RPE: 9-10 for weeks 1-4, RPE 11-12 for weeks 4-8, and RPE 13-14 for weeks 9-12, target heart rate of 30-60% heart rate reserve during exercise, 50 min | Supervised | 12 weeks | 3 | HG (dynamometer) |
| Haykowsky et al., 2005 [104] | 31 | healthy | EX_1_:66.0±3.0  EX_2_:70.0±4.0  EX_3_:68.0±6.0  CON:67.0±4.0 | ND | EX_1_: Aerobic  EX_2_: Resistance  EX_3_: Combined | EX_1_: 15-42.5 min at 60-80% of HRR  EX_2_: whole body exercises; 2 sets of 10 reps at 50–75% of 1RM  EX_3_: the combined group performed same programs of aerobic and resistance described | Supervised | 12 weeks | 3 | VO_2peak_ (cycling test-gas analyzer), UBS, LBS (1RM) |
| Holsgaard-Larsen et al., 2011 [105] | 23 | healthy | EX:69.7±3.4  CON:69.7±3.4 | ND | Resistance | Lower body exercises; 4 sets of 8-10 reps at 75–80% of 1RM | Supervised | 12 weeks | 2 | LBS (isokinetic dynamometer) |
| Jang and Park. 2021 [106] | 20 | healthy | EX:73.3±4.5  CON:71.9±6.7 | ND | Resistance | Lower body exercises; 1-2 sets of 10 reps | ND | 4 weeks | 3 | HG (dynamometer) |
| Janzen et al., 2006 [107] | 57 | healthy | EX_1_:54.8±6.5  EX_2_:55.8±8.2  CON:58.8±6.7 | ND | EX_1_: Bilateral resistance  EX_2_: Unilateral resistance | EX_1_: whole body bilateral resistance exercises; 1-2 sets of 8-12 reps at 50-60% of 1RM  EX_2_: whole body unilateral resistance exercises; 1-2 sets of 12 reps at 50-60% of 1RM | Supervised | 26 weeks | 3 | UBS, LBS (1RM) |
| Judge et al., 1993 [108] | 38 | fall-associated injuries | EX:67.8±2.8  CON:68.5±4.1 | EX:22.8±3.6  CON:26.8±4.8 | Combined | Lower body exercises; 3 sets of 10-14 reps at 70% of 1RM + 20-min at 70% of HR_max_ + balance | Supervised | 6 months | 3 | LBS (1RM) |
| Kallinen et al., 2002 [109] | 42 | healthy | EX_1_:76-78  EX_2_:76-78  CON:76-78 | ND | EX_1_: Aerobic  EX_2_: Resistance | EX_1_: 20-40 min at 50-80% of HRR  EX_2_: whole body exercises; 3-4 sets of 8-10 reps at 60-75% of 1RM | Supervised | 18 weeks | 2-3 | VO_2max_ (cycling test) |
| Kim et al., 2016 [110] | 69 | sarcopenic obesity | EX:81.4±4.3  CON:81.1±5.1 | EX:25.1±2.5  CON:25.3±2.8 | Combined | whole body elastic band, chair, and machine exercises; 1-3 sets of 10 reps | Supervised | 3 months | 2 | HG, LBS (isometric dynamometer) |
| Kim et al., 2015 [111] | 66 | community-dwelling frail | EX:81.1±2.8  CON:80.3±3.3 | ND | Resistance | whole body elastic band exercises; with 8 reps at a rating of 12-14 BRPE + 20 min of balance and gait training | Supervised | 3 months | 2 | HG, LBS (isometric dynamometer) |
| Kim et al., 2013 [112] | 74 | non-specific knee pain | EX:80.9±2.3  CON:80.5±2.7 | EX:23.7±3.2  CON:23.6±3.1 | Resistance | lower body elastic band exercises; with 8 reps at a rating of 12-14 BRPE + 20 min of balance and gait training | Supervised | 3 months | 2 | HG (isometric dynamometer) |
| Kim et al., 2014 [113] | 105 | history of falls | EX:77.8±4.2  CON:77.8±4.2 | EX:21.3±3.1  CON:23.6±3.2 | Resistance | whole body exercises; with 8 reps at a rating of 12-14 BRPE + 20 min balance | ND | 3 months | 2 | HG, LBS (isometric dynamometer) |
| Korpelainen et al., 2006 [114] | 160 | low bone mineral density | EX:72.9±1.1  CON:72.8±1.2 | EX:25.7±3.4  CON:25.5±3.5 | Combined | strength, balance, and jumping exercises, including walking, walking with arm movements, knee bends, leg lifts, heel rises and drops, dancing, stamping, stepping sideways, forward, and backward, stair climbing, stepping up and down from benches, and jumping for 45-min + 20 min at home (daily) | Supervised & unsupervised | 30 months | 1-7 | HG, LBS (isometric dynamometer) |
| Kwon et al., 2015 [115] | 59 | healthy | EX:77.0±4.2  CON:76.9±3.9 | ND | Resistance | body weight exercises as well as exercises using Thera bands, dumbbells, and balls; 1 set of 5-10 reps | Supervised | 12 weeks | 1 | HG (dynamometer) |
| Letieri et al., 2018 [116] | 39 | healthy | EX_1_:66.8±4.4  EX_2_:71.3±4.7  CON:69.0±6.4 | EX_1_:29.7±4.8  EX_2_:28.5±3.7  CON:29.6±1.8 | EX_1_: High intensity resistance  EX_2_: Low intensity resistance | EX_1_: lower body exercises; 3-4 sets of 6-8 reps at 70-80% of 1RM  EX_2_: ND | Supervised | 16 weeks | 3 | LBS (isokinetic dynamometer) |
| Liao et al., 2018 [117] | 56 | sarcopenic obesity | EX:66.7±4.5  CON:68.3±6.1 | EX:27.3±3.7  CON:29.2±3.6 | Resistance | whole body elastic band exercises; 3 sets of 10 reps at a rating of 13 BRPE | Supervised | 12 weeks | 3 | HG, LBS (isometric dynamometer) |
| Liu-Ambrose et al., 2004 [118] | 68 | low bone mass | EX:79.6±2.1  CON:79.5±3.2 | ND | Resistance | whole body exercises; 1-2 sets of 6-15 reps at 50-85% of 1RM | Supervised | 25 weeks | 2 | LBS (ND) |
| Lord et al., 1995 [119] | 197 | older | EX:71.6±5.5  CON:71.7±5.3 | ND | Combined | aerobic exercises, strengthening exercises, and activities for balance, flexibility, endurance, and hand-eye and foot-eye coordination | ND | 12 months | 2 | LBS (using a strap assembly) |
| Lu et al., 2013 [120] | 31 | healthy | EX:73.9±6.6  CON:68.9±5.8 | EX:24.6±3.1  CON:24.8±3.3 | Tai chi | 12-forms Yang-style Tai Chi for 60-min | Supervised | 16 weeks | 3 | LBS (dynamometer) |
| Maddalozzo et al., 2007 [121] | 69 | healthy | EX:52.3±3.3  CON:52.5±3.0 | ND | Resistance | resistance exercises; 3 sets of 8-12 reps at 60-75% of 1RM | Supervised | 52 weeks | 2 | UBS, LBS (isokinetic dynamometer) |
| Malandish et al., 2020 [122] | 29 | vitamin D deficiency | EX:54.4±4.0  CON:54.0±3.3 | EX:28.6±4.2  CON:30.4±6.6 | Aerobic | 50-60 min at 65-70% of HRR | Supervised | 12 weeks | 3 | VO_2max_ (treadmill test) |
| Marcos-Pardo et al., 2019 [123] | 27 | healthy | EX:65-75  CON:65-75 | EX:27.6±4.4  CON:26.1±3.3 | Resistance | whole body exercises; 8-12 reps,  60-80% of 1RM | Supervised | 12 weeks | 3 | UBS, LBS (1RM) |
| Marcus et al., 2009 [124] | 16 | impaired glucose tolerance | EX:56.3±6.4  CON:53.2±6.5 | EX:28.5±3.7  CON:32.2±4.0 | Resistance | lower extremity extensor muscle contractions at very light to somewhat hard | Supervised | 12 weeks | 3 | LBS (isometric dynamometer) |
| Marín-Cascales et al., 2015 [125] | 40 | healthy | EX:57.7±7.1  CON:62.4±5.1 | EX:29.3±3.9  CON:29.4±4.7 | Multi component exercise | small reactive vertical jumps from 4x10 drop jumps to 6x10 +30-45 min at 50-60% of HRR | ND | 12 weeks | 3 | LBS (isokinetic dynamometer) |
| Marques et al., 2011 (a) [126] | 60 | healthy | EX:70.1±5.4  CON:68.2±5.7 | EX:28.4±3.7  CON:28.2±3.7 | Combined | 60-min multi-component exercises;  consisting of marching in place, stepping exercise at a speed of 120-125 beats per minute, resistance, balance, and agility | Supervised | 8 months  (32weeks) | 2 | LBS (isokinetic dynamometer), HG (dynamometer) |
| Marques et al., 2011 (b) [127] | 71 | healthy | EX_1_:70.3±5.5  EX_2_:67.3±5.2  CON:67.9±5.9 | EX_1_:27.5±3.8  EX_2_:28.8±4.6  CON:28.1±3.5 | EX_1_: Aerobic EX_2_: Resistance | A: 35-40 min at 50-85% of HRR  R: whole body exercises; 2 sets of 8-10 reps at 60-80% of 1RM | Supervised | 8 months (32 weeks) | 3 | LBS (isokinetic dynamometer) |
| Mazini Filho et al., 2018 [128] | 65 | healthy | EX:60-75  CON:60-75 | EX:27.0±4.5  CON:25.5±13.1 | Resistance | whole body exercises; 2-3 sets of 8-15 reps at a rating of 4-8 on the OMNI scale | ND | 12 weeks | 3 | LBS (10RM) |
| Miura et al., 2008 [129] | 77 | healthy | EX_1_:69.0±6.5  EX_2_:69.5±7.0  CON:68.9±7.5 | EX_1_:22.8±2.4  EX_2_:23.5±2.7  CON:23.7±3.0 | Resistance | Circuit exercises; 3-5 sets of 15-20 reps of each exercise. chair-based exercises, women performed various types of leg exercises in a four-beat. Each exercise was repeated up to 16 times while cycling for 20 min. | Supervised | 12 weeks | EX_1_: 1  EX_2_: 2 | HG (dynamometer) |
| Moreira et al., 2013 [130] | 108 | healthy | EX:58.6±6.7  CON:59.3±6.1 | ND | Water-based exercise | aerobic water based at 55% of HR_max_ + whole body water based exercises; 2-5 sets of 10-30 s exercises at a rating of 6-9 BRPE | ND | 24 weeks | 3 | LBS (isometric dynamometer) |
| Morganti et al., 1995 [131] | 40 | healthy | EX:61.1+3.6  CON:57.3+6.1 | EX:24.4±4.9  CON:23.1±2.2 | Resistance | whole body exercises; 3 sets of 8 reps at 50-80% of 1RM | Supervised | 12 months | 2 | UBS, LBS (1RM) |
| Multanen et al., 2014 [132] | 80 | mild knee osteoarthritis | EX:58.0±4.0  CON:59.0±4.0 | EX:27.1±3.1  CON:26.7±4.2 | Aerobic | 55-min of multidirectional exercise including aerobic and step‐aerobic jumping exercise programs, alternated every two weeks | Supervised | 12 months | 3 | LBS (isometric dynamometer),  VO_2max_ (2-km walk test) |
| Nicholson et al., 2015 [133] | 57 | healthy | EX:66.0±4.1  CON:65.6±4.7 | EX:26.0±3.2  CON:24.5±2.9 | Resistance | whole body very high repetition resistance exercises | Supervised & unsupervised | 6 months | 2 | UBS, LBS (1RM) |
| Nunes et al., 2016 [134] | 38 | healthy | EX_1_:62.7±6.3  EX_2_:60.7±9.1  CON:59.5±8.7 | EX_1_:27.9±0.7  EX_2_:28.1±8.7  CON:30.4±7.0 | Resistance | EX_1_: whole body exercises; 3 sets of 8-12 reps at 70% of 1RM  EX_2_: whole body exercises; 6 sets of 8-12 reps at 70% of 1RM | Supervised | 16 weeks | 3 | LBS (1RM) |
| Oh et al., 2021 [135] | 60 | knee osteoarthritis | EX:72.4±6.3  CON:71.1±5.4 | EX:24.8±2.5  CON:25.7±3.8 | Resistance | whole body chair and elastic band exercises | Supervised & unsupervised | 5 months | 2-3 | HG, LBS (isometric dynamometer) |
| Oh et al., 2017 [136] | 80 | community dwelling | EX:74.9±6.5  CON:73.5±2.2 | EX:24.8±2.6  CON:25.0±2.6 | Resistance | whole body elastic band exercises; 2-3 sets of 10-20 reps | Supervised & unsupervised | 18 weeks | 2 | LBS (isokinetic dynamometer) |
| Oliveira et al., 2018 [137] | 34 | healthy | EX:55.5±6.8  CON:54.1±5.2 | EX:27.2±2.7  CON:27.3±2.4 | Pilates | 21 strengthening and stretching exercises; at a rating of 5-6 BRPE for 60-min | Supervised | 6 months | 3 | LBS (isokinetic dynamometer) |
| Oliveira et al., 2017 [138] | 32 | healthy | EX:63.6±1.0  CON:64.2±0.8 | EX:24.7±1.3  CON:25.0±1.2 | Pilates | 20 strengthening and stretching exercises; at a rating of 5-6 BRPE for 60-min | Supervised | 12 weeks | 2 | LBS (isokinetic dynamometer) |
| Oliveira et al., 2017 [139] | 30 | healthy | EX:65.1±3.7  CON:66.3±3.4 | EX:27.4±5.0  CON:28.2±7.5 | Pilates | 19 strengthening and stretching exercises; at a rating of 5-6 BRPE for 60-min | Supervised | 12 weeks | 2 | UBS (isokinetic dynamometer) |
| Orsatti et al., 2008 [140] | 50 | healthy | EX:57.8±8.0  CON:59.3±6.2 | EX:28.8±4.5  CON:27.6±5.1 | Resistance | whole body exercises; 1-3 sets of 8-15 reps at 40-80% of 1RM | Supervised | 16 weeks | 3 | UBS, LBS (1RM) |
| Paolillo et al., 2014 [141] | 30 | healthy | EX:55.0±2.0  CON:55.0±2.0 | EX:27.0±4.0  CON:33.0±7.0 | Aerobic | 45-min at 85-90% of HR_max_ | Supervised | 6 months | 2 | LBS (isokinetic dynamometer) |
| Park et al., 2017 [142] | 50 | sarcopenic obesity | EX:73.5±7.1  CON:74.7±5.1 | EX:27.0±1.4  CON:27.6±2.0 | Combined | A: 30-50 min at a rating of 13-17 BRPE  R: whole body elastic band exercises; 2-3 sets of 8-15 reps at 80% of 1RM | Supervised | 24 weeks | A: 5  R: 3 | HG (dynamometer) |
| Park et al., 2021 [143] | 54 | early knee osteoarthritis | EX:66.9±4.6  CON:68.0±4.2 | ND | Resistance | Lower isometric exercises; at 60-80% of maximum tolerance or rating of 10-15 BRPE | ND | 8 weeks | 3 | LBS (isokinetic dynamometer) |
| Parkhouse et al., 2000 [144] | 22 | low bone mineral density | EX:67.0±1.0  CON:70.0±2.0 | EX:22.0±2.0  CON:25.0±4.0 | Resistance | Lower body exercises; 3 sets of 8-10 reps at 75-80% of 1RM | ND | 8 months | 3 | LBS (1RM) |
| Porter, et al., 2002 [145] | 28 | elderly | EX:60±4.0  CON:58±6.0 | EX:23.6±2.5  CON:23.0±2.2 | Resistance | whole body exercises; 3 sets of 8 reps at 80% of 1RM | Supervised | 2 years | 2 | UBS, LBS (1RM) |
| Pruitt et al., 1995 [146] | 40 | healthy | EX_1_:67.0±0.5  EX_2_:67.6±1.4  CON:69.6±4.2 | EX_1_:24.5±3.4  EX_2_:23.9±1.6  CON:25.1±3.1 | Resistance | EX_1_: whole body exercises; 3 sets of 7-14 reps at 40-80% of 1RM  EX_1_: whole body exercises; 3 sets of 14 reps at 40% of 1RM | Supervised | 12 months | 3 | UBS (1RM) |
| Pu et al., 2001 [147] | 18 | chronic heart failure | EX:76.6±6.0  CON:76.6±6.3 | EX:24.7±3.6  CON:28.0±5.0 | Resistance | whole body exercises; 3 sets of 8 reps at 80% of 1RM | Supervised | 10 weeks | 3 | UBS, LBS (1RM), VO_2peak_ (treadmill test-gas analyzer) |
| Ramírez-Campillo et al., 2014 [148] | 60 | healthy | EX_1_:66.3±3.7  EX_2_:68.7±6.4  CON:66.7±4.9 | EX_1_:31.7±5.0  EX_2_:31.0±6.5  CON:29.5±3.0 | Resistance | EX_1_: whole body exercises; 3 sets of 8 reps at 40-75% of 1RM with high speed movements  EX_2_: whole body exercises; 3 sets of 8 reps at 75% of 1RM with low speed movements | Supervised | 12 weeks | 3 | UBS, LBS (1RM), HG (dynamometer) |
| Ramirez-Campillo et al., 2016 [149] | 24 | healthy | EX_1_:70.0±6.9  EX_2_:71.9±6.3  CON:68.9±7.5 | EX_1_:28.0±4.1  EX_2_:29.6±3.4  CON:27.4±4.0 | Resistance | whole body exercises; with 8 reps at 75% of 1RM | Supervised | 12 weeks | EX_1_: 2  EX_2_: 3 | HG (dynamometer) |
| Reis et al., 2012 [150] | 58 | sedentary | EX:52.9±4.0  CON:53.9±5.1 | ND | Resistance | Lower body exercises; 2-4 sets of 6-15 reps at 60-85% of 1RM | ND | 3 months | 2 | LBS (1RM) |
| Rezende et al., 2016 [151] | 44 | nonalcoholic fatty liver disease | EX:56.2±7.8  CON:54.5±8.9 | EX:34.1±4.4  CON:32.0±5.0 | Aerobic | 30-50 min at ventilatory anaerobic  threshold up to 10% below respiratory compensation point | Supervised | 24 weeks | 2 | VO_2max_ (treadmill test-gas analyzer) |
| Rhodes et al., 2000 [152] | 44 | healthy | EX:68.8±3.2  CON:68.2±3.5 | ND | Resistance | whole body exercises; 3 sets of 8 reps at 75% of 1RM | Supervised | 12 months | 3 | UBS, LBS (1RM), HG (dynamometer) |
| Ribeiro et al., 2017 [153] | 76 | healthy | EX_1_:69.7±6.6  EX_2_:68.9±5.8  CON:66.8±4.2 | EX_1_:28.0±5.2  EX_2_:27.3±4.6  CON:26.3±4.6 | Resistance | EX_1_: whole body exercises; 3 sets of 8-12 reps with the same load in all three sets  EX_2_: whole body exercises; 3 sets of 8-12 reps with the load increasing and number of repetitions decreasing for each set | Supervised | 8 weeks | 3 | UBS, LBS (1RM) |
| Rodrigues-Krause et al., 2018 [154] | 20 | healthy | EX:64.0±2.1  CON:66.0±6.3 | EX:28.2±3.7  CON:27.7±3.6 | Aerobic | 40-min at 50-60% of VO_2peak_ | Supervised | 8 weeks | 3 | VO_2max_ (treadmill test-gas analyzer), LBS (isokinetic dynamometer) |
| Roelants et al., 2004 [155] | 59 | healthy | EX:63.9±0.8  CON:64.2±0.6 | EX:26.9±0.8  CON:26.4±1.2 | Resistance | Lower body exercises; 2-3 sets at 8-15 RM | Supervised | 24 weeks | 3 | LBS (isokinetic dynamometer) |
| Santos et al., 2019 [156] | 27 | breast cancer survivors | EX:55.0±5.8  CON:54.3±5.2 | EX:28.0±5.0  CON:26.8±4.0 | Resistance | whole body exercises; 3 sets of 8-12 reps | Supervised | 8 weeks | 1 | UBS, LBS (10RM) |
| Seo et al., 2021 [157] | 27 | sarcopenia | EX:70.3±5.4  CON:72.9±4.8 | EX:22.9±2.0  CON:22.4±1.5 | Resistance | whole body weight-based and elastic band; 3-5 sets of 6-15 reps at a rating of 4-8 on the OMNI Scale | Supervised | 16 weeks | 3 | HG (dynamometer) |
| Shaw et al., 2016 [158] | 37 | healthy | EX:60.4±5.3  CON:57.7±2.8 | EX:26.1±3.1  CON:24.5±3.9 | Resistance | whole body exercises; 3 sets of 8-10 reps at 67-85% of 1RM | ND | 6 weeks | 2 | UBS, LBS (1RM), VO_2max_ (6 min walking test) |
| Son et al., 2017 [159] | 20 | hypertension | EX:76.0±15.8  CON:74.7±6.3 | EX:22.8±2.2  CON:24.1±0.6 | Combined | A: 30-min at 40-70% of HRR  R: whole body resistance band exercises for 20 min | Supervised | 12 weeks | 3 | VO_2max_ (Bruce) |
| Song et al., 2003 [160] | 72 | osteoarthritis | EX:64.8±6.0  CON:62.5±5.6 | EX:24.9±2.6  CON:26.4±3.5 | Tai chi | 12 main movements of Tai chi with Qigong  breathing exercise + every day at home 20 min | Supervised & unsupervised | 12 weeks | 3-5 | LBS (isokinetic dynamometer) |
| Song et al., 2010 [161] | 82 | osteoarthritis | EX:63.0±7.3  CON:61.2±8.0 | ND | Tai chi | 31 forms of Sun-style Tai chi with Qigong  breathing exercise + every day at home 20 min | Supervised | 6 months | every day at home | LBS (dynamometer) |
| Souza et al., 2017 [162] | 45 | healthy | EX:67.3±4.3  CON:67.1±4.5 | EX:26.8±5.1  CON:26.6±4.7 | Resistance | whole body exercises; 3 sets of 10-15 reps | ND | 12 weeks | 3 | UBS, LBS (1RM) |
| Speck et al., 2010 [163] | 295 | breast cancer survivors (lymphedema) | EX:56.0±9.0  CON:58.0±9.0 | ND | Resistance | whole body exercises; 3 sets of 10 reps | Supervised | 1 year | 2 | UBS, LBS (1RM) |
|  |  | breast cancer survivors (no lymphedema) | EX:55.0±7.0  CON:57.0±8.0 |  |  |  |  |  |  |  |
| Stanghelle et al., 2020 [164] | 149 | osteoporosis and vertebral fracture | EX:74.7±6.1  CON:73.7±5.6 | EX:23.2±3.4  CON:23.2±4.1 | Resistance | multicomponent exercise; 2 series with 8-12 reps at a rating of 13 -14 BRPE | Supervised | 12 weeks | 2 | HG (dynamometer) |
| Stojanović et al., 2021 [165] | 180 | healthy | EX:75.7±8.9  CON:74.5±8.2 | ND | Resistance | whole body elastic band exercises; 2 sets of 12-15 reps at 40-60% of 1RM | Supervised | 12 weeks | 2 | HG (dynamometer) |
| Strandberg et al., 2015 [166] | 42 | healthy | EX:68.0±2.0  CON:68.0±1.0 | EX:24.5±2.8  CON:24.5±2.8 | Resistance | whole body exercises; 3 sets of 8-15 reps at 75-85% of 1RM | Supervised | 24 weeks | 2 | LBS (1RM) |
| Taaffe et al., 1996 [167] | 36 | healthy | EX_1_:67.2±0.6  EX_2_:67.2±0.4  CON: 69.6±1.3 | EX_1_:24.9±1.0  EX_2_:24.3±0.5  CON:25.1±0.9 | Resistance | EX_1_: whole body exercises; 3 sets of 7 reps at 70-80% of 1RM  EX_2_: whole body exercises; 3 sets of 14 reps at 35-40% of 1RM | Supervised | 52 weeks | 3 | LBS (1RM) |
| Takeshima et al., 2002 [168] | 30 | healthy | EX:69.3±4.5  CON:69.3±3.3 | ND | Water-based exercise | 30 min water-based endurance-type exercise + whole body water-based resistance exercises; 1 set of 10-15 reps | Supervised | 12 weeks | 3 | VO_2max_ (cycling test-gas analyzer) |
| Timonen et al., 2002 [169] | 68 | frail older | EX:83.5±4.1  CON:82.6±3.7 | ND | Resistance | Lower body exercise; 2 sets of 8-20 reps + 30 min functional training; 2 sets of 15 reps | Supervised | 10 weeks | 2 | LBS (isometric) |
| Tomeleri et al.,2020 [170] | 54 | healthy | EX_1_:71.4±6.0  EX_2_:69.7±5.7  CON: 68.6±5.1 | EX_1_:27.4±4.0  EX_2_:26.7±4.5  CON: 26.8±3.7 | Resistance | EX_1_: multi-joint to single-joint order of resistance training; 3 sets of 10-15 reps at 60% of 1RM  EX_1_: single-joint to multijoint order of resistance training; 3 sets of 10-15 reps at 60% of 1RM | Supervised | 12 weeks | 3 | UBS, and LBS (1RM) |
| Tsourlou et al., 2006 [171] | 24 | healthy | EX:69.3±6.6  CON:68.4±6.7 | EX:28.2±2.8  CON:29.3±3.5 | Water-based exercise | 25-min aerobic water-based exercise program at 65-80% of HR_max_ + whole body resistance exercises with specialized water-resistance equipment; 2-3 sets of 12-15 reps | Supervised | 24 weeks | 3 | UBS, and LBS (3RM)**,** HG (dynamometer) |
| Tsutsumi et al., 1998 [172] | 36 | healthy | EX_1_:68.5±6.1  EX_2_:68.5±6.1  CON:68.5±6.1 | ND | Resistance | EX_1_: whole body elastic band exercises; 2 sets of 8-10 reps at 75-85% 1RM  EX_2_: whole body elastic band exercises; 2 sets of 14-16 reps at 55-65% 1RM | ND | 12 weeks | 3 | UBS, and LBS (1RM) |
| Urzi et al., 2019 [173] | 35 | living in a nursing home | EX:84.4±7.7  CON:88.9±5.3 | EX:28.0±5.5  CON:29.1±5.1 | Resistance | whole body elastic band exercises; at a rating of 12-14 BRPE | Supervised | 12 weeks | 3 | HG (dynamometer) |
| Uusi-Rasi et al., 2015 [174] | 205 | older | EX:74.8±2.9  CON:73.8±3.1 | ND | Resistance | balance challenging, weight bearing, strengthening, agility, and functional exercises at 30-75% of 1RM. | Supervised | 2 years | 1-2 | LBS (isometric) |
| Valkeinen et al., 2008 [175] | 26 | fibromyalgia | EX:59.0±3.0  CON:58.0±3.0 | EX:27.0±2.0  CON:28.0±4.0 | Combined | A: 30-60 min at aerobic and anaerobic thresholds  R: whole body exercises; 2 sets of 5-20 reps at 40-80% of 1RM | Supervised & unsupervised | 21 weeks | 4-6 | VO_2peak_ (gas analyzer)**,** LBS (1RM), HG (dynamometer) |
| Valkeinen et al., 2005 [176] | 26 | fibromyalgia | EX:60.0±2.0  CON:59.0±4.0 | ND | Resistance | whole body exercises; at 40-80% of 1RM | Supervised | 21 weeks | 2 | LBS(1RM) |
| van Gemert et al., 2015 [177] | 146 | overweight and obese | EX:59.5±4.9  CON:60.0±4.9 | EX:29.0±2.9  CON:29.5±2.6 | Combined | A: 60-min at 60-95% of HRR  R: strength training | Supervised | 16 weeks | 4 | VO_2peak_ (maximal cycle exercise test) |
| Vasconcelos et al., 2020 [178] | 48 | older | EX_1_:64.2±4.7  EX_2_:64.9±3.0  CON:65.9±5.8 | EX_1_:29.6±4.4  EX_2_:29.6±5.0  CON:31.8±5.8 | EX_1_: Functional  EX_2_: Combined | EX_1_: 15-min agility, coordination, and muscle power at a rating of 6-7 on the OMNI scale + 20 min multi-articular exercises for lower and upper limbs at a rating of 7-8 on the OMNI scale  EX_2_: 30 s running and 30 s walking at a rating of 6-7 on the OMNI scale + whole body resistance exercises at a rating of 7-8 on the OMNI scale | Supervised | 26 weeks | 3 | HG (dynamometer) |
| Vasconcelos et al., 2016 [179] | 31 | sarcopenic obesity | EX:72.0±4.6  CON:72.0±3.6 | EX:32.0±2.3  CON:33.0±2.9 | Resistance | Lower body exercise; 2-3 sets of 8-12 reps at 40-75% of 1RM | Supervised | 10 weeks | 2 | LBS (isokinetic) |
| Vassão et al., 2020 [180] | 34 | knee osteoarthritis | EX:61.7±4.3  CON:65.4±4.2 | EX:30.0±3.4  CON:27.5±3.3 | Resistance | Lower body exercise; 3 sets with 8 reps at 60% of 1RM | Supervised | 8 weeks | 2 | LBS (isometric) |
| Vélez-Toral et al., 2017 [181] | 166 | healthy | EX:56.2±4.1  CON:55.7±4.0 | EX:27.0±3.7  CON:27.3±4.9 | Combined | aerobic cardiorespiratory fitness, muscle resistance, and other fitness functions | Supervised | 20 weeks | 3 | HG (dynamometer) |
| Venturelli et al., 2010 [182] | 36 | older | EX:83.3±6.7  CON:84.1±5.8 | ND | Resistance | Upper body circuit exercises; 3 series with 20 reps started at 50% of 1RM | Supervised | 12 weeks | 3 | UBS (1RM) |
| Verschueren et al., 2004 [183] | 46 | healthy | EX:63.9±3.8  CON:64.2±3.1 | EX:27.4±3.5  CON:26.5±5.8 | Resistance | Lower body exercise; 2-3 sets of 10-15 reps | ND | 24 weeks | 3 | LBS (isokinetic dynamometer) |
| von Stengel et al., 2012 [184] | 101 | older | EX:68.6±3.0  CON:68.1±2.7 | EX:26.2±4.2  CON:27.5±5.0 | Combined | A: 20-min at 70-80% of HR_max_  R: 35-min strength training | Supervised | 18 months | 2 | LBS (isometric dynamometer) |
| Widjaja et al., 2021 [185] | 34 | overweight and obese | EX:64.0±3.0  CON:61.0±5.0 | EX:28.0±2.1  CON:28.4±4.6 | Thai yoga | 30 min Thai Yoga training | ND | 8 weeks | 3 | LBS (isometric dynamometer) |
| Wong et al., 2018 (a) [186] | 44 | stage 2 hypertension | EX:59.0±4.5  CON:59.0±4.6 | EX:24.2±3.6  CON:23.8±3.7 | Aerobic | 2-5 ×12 sets of 192 stairs climbing at a rating of 11-13 BRPE by 5 min recovery | Supervised | 12 weeks | 4 | LBS (8RM) |
| Wong et al., 2018 (b) [187] | 100 | stage 2 hypertension | EX:74.0±4,0  CON:73.0±4.0 | EX:26.0±2.8  CON: 26.9±2.9 | Water-based exercise | 25-45 min at 60-75% of HR_max_ | Supervised | 20 weeks | 3-4 | HG (dynamometer), VO_2max_ (Bruce) |
| Wooten et al., 2011 [188] | 24 | obese | EX:64.4±2.7  CON:67.0±2.1 | EX:31.0±1.5  CON:34.0±4.5 | Resistance | Whole body exercises; 3 sets (2 sets at 8-RM and 1 set to failure) | Supervised | 12 weeks | 3 | UBS, and LBS (8RM) |
| Yoo et al., 2010 [189] | 28 | healthy | EX:70.9±2.7  CON:71.1±2.7 | EX:26.6±2.9  CON:25.4±3.0 | Aerobic | 45-min at 60% of HRR | Supervised | 12 weeks | 3 | HG (dynamometer) |
| Yoon et al., 2017 [190] | 58 | mild cognitive impairment | EX_1_:75.0±0.9  EX_2_:76.0±1.3  CON:78.0±1.0 | EX_1_: 25.5±2.5  EX_2_: 23.9±3.7  CON: 22.9±1.8 | Resistance | EX_1_: elastic band exercises; 2-3 sets of 12-15 reps at a rating of 12-13 BRPE  EX_2_: elastic band exercises; 2-3 sets of 8-10 reps at a rating of 15-16 BRPE | Supervised | 12 weeks | 2 | LBS (isokinetic dynamometer), HG (dynamometer) |
| **Abbreviations:** lower-body muscular strength (LBS), upper-body muscular strength (UBS), handgrip strength (HG), maximal/peak oxygen uptake (VO_2max/peak_), maximal/peak heart rate (HR_max/peak_), one repetition maximum (1RM), Borg rating of perceived exertion (BRPE), repetitions (reps), computed tomography scan (CT), Dual-energy X-ray absorptiometry (DEXA), not-described (ND) | | | | | | | | | | |

**Supplementary datasheet 1.** Summary of demographic characteristics of participants and interventions
