# Supplementary material for: Influence of exercise type and duration on cardiorespiratory fitness and muscular strength in post-menopausal women: a systematic review and meta-analysis
Source: Front Cardiovasc Med. 2023 May 9;10:1190187. doi: 10.3389/fcvm.2023.1190187 (PMC10204927; doi:10.3389/fcvm.2023.1190187)
Supplement: Supplementary file 2 [file Table2.docx]

**Supplementary Table 2. Summary of subgroup analyses**

|  |  | | Moderators | N | SMD (95% CI) | P-value | P-heterogeneity |
| --- | --- | --- | --- | --- | --- | --- | --- |
| **CRF** | | Age (year) | Middle<65 | 16 | 1.06 (0.63 to 1.48) | 0.001 | 0.001 |
|  |  |  | Elderly≥65 | 19 | 1.24 (0.78 to 0.70) | 0.001 | 0.001 |
|  |  | Intervention duration | Medium-term | 25 | 1.17 (0.80 to 1.55) | 0.001 | 0.001 |
|  |  |  | Long-term | 10 | 1.12 (0.53 to 1.71) | 0.001 | 0.001 |
|  |  | Type of exercise | Aerobic | 12 | 1.21 (0.68 to 1.74) | 0.001 | 0.001 |
|  |  |  | Combined | 7 | 1.47 (0.81 to 2.12) | 0.001 | 0.01 |
|  |  |  | Resistance | 8 | 1.26 (0.34 to 2.28) | 0.007 | 0.001 |
|  |  |  | water-based | 4 | 0.83 (0.17 to 1.49) | 0.01 | 0.006 |
| **Lower-body muscular strength** | | Age (year) | Middle<65 | 36 | 0.98 (0.74 to 1.22) | 0.001 | 0.001 |
|  |  |  | Elderly≥65 | 72 | 1.03 (0.83 to 1.22) | 0.001 | 0.001 |
|  |  | Intervention duration | Medium-term | 60 | 1.30 (1.03 to 1.58) | 0.001 | 0.001 |
|  |  |  | Long-term | 49 | 0.82 (0.64 to 1.00) | 0.001 | 0.001 |
|  |  | Type of exercise | Aerobic | 7 | 0.49 (0.18 to 0.81) | 0.002 | 0.28 |
|  |  |  | Combined | 10 | 0.61 (0.32 to 0.91) | 0.001 | 0.004 |
|  |  |  | Resistance | 78 | 1.28 (1.50 to 11.64) | 0.001 | 0.001 |
|  |  |  | Water-based | 5 | 0.96 (0.10 to 1.83) | 0.02 | 0.001 |
| **Upper-body muscular strength** | | Age (year) | Middle<65 | 15 | 1.02 (0.75 to 1.30) | 0.001 | 0.002 |
|  |  |  | Elderly≥65 | 40 | 1.15 (0.88 to 1.41) | 0.001 | 0.001 |
|  |  | Intervention duration | Medium-term | 38 | 1.13 (0.85 to 1.41) | 0.001 | 0.001 |
|  |  |  | Long-term | 17 | 1.08 (0.82 to 1.34) | 0.001 | 0.001 |
|  |  | Type of exercise | Resistance | 43 | 1.20 (0.97 to 1.43) | 0.001 | 0.001 |
|  |  |  | Water-based | 5 | 0.30 (-0.08 to 0.69) | **0.12** | 0.48 |
| **Handgrip strength** | | Age | Middle<65 | 8 | 1.62 (1.12 to 2.11) | 0.001 | 0.001 |
|  |  |  | Elderly≥65 | 41 | 2.49 (0.72 to 4.25) | 0.006 | 0.001 |
|  |  | Intervention duration | Medium-term | 33 | 1.83 (1.07 to 2.59) | 0.001 | 0.001 |
|  |  |  | Long-term | 16 | 1.82 (1.16 to 2.47) | 0.001 | 0.09 |
|  |  | Type of exercise | Aerobic | 4 | 1.87 (-0.77 to 4.52) | 0.16 | 0.001 |
|  |  |  | Combined | 9 | 1.82 (0.94 to 2.69) | 0.001 | 0.03 |
|  |  |  | Resistance | 30 | 1.72 (1.00 to 2.44) | 0.001 | 0.002 |
